# Symptom-Level Precision Neurology in Amyotrophic Lateral Sclerosis (ALS): Linking Microglial Pruning, Mitochondrial Nicotinamide Adenine Dinucleotide (NAD+) Compensation, and Autophagy Failure Across the Aging Spectrum

**DOI:** 10.7759/cureus.109147

**Published:** 2026-05-18

**Authors:** Ngo Cheung

**Affiliations:** 1 Psychiatry, Cheung Ngo Medical Limited, Hong Kong, HKG

**Keywords:** als–ftd spectrum, amyotrophic lateral sclerosis, autophagy, microglial pruning, mitochondrial dysfunction, nad+, precision neurology, synaptic plasticity

## Abstract

Amyotrophic lateral sclerosis (ALS) is a heterogeneous neurological disease with limited disease-modifying treatment options and, for many patients, a short survival window. The clinical course varies widely. Limb weakness, bulbar impairment, respiratory decline, fine-motor dysfunction, cognitive change, mood symptoms, and fatigue may each appear at different times and progress at different rates. This variability suggests that motor neuron loss alone may not fully explain the patient-level pattern of symptoms. This article is a narrative hypothesis framework, not a clinical guideline or a validated stratification tool. Established ALS biology, associative genomic findings, preclinical observations, computational predictions, and author-derived hypotheses are therefore separated throughout the article. This review brings together four interlinked studies by the current author as a primary hypothesis-generating corpus, which proposes that synaptic plasticity fragility may initiate a microglial pruning continuum shared by major depressive disorder and ALS, while ALS-specific progression may depend on mitochondrial stress, oxidized nicotinamide adenine dinucleotide (NAD+) compensation failure, and collapse of autophagy under aging-related limits. The model presented here maps symptom domains to vulnerable circuit compartments and separates three broad biological states: compensated plasticity, fragile plasticity, and network collapse. A compact mechanistic formulation is used to describe the balance between pruning pressure, glutamatergic burden, and aging stress on one side, and oxidative phosphorylation capacity, NAD+ reserve, and autophagic clearance on the other. The framework also incorporates opposing phosphoinositide 3-kinase (PI3K)/AKT/mechanistic target of rapamycin (mTOR) and peroxisome proliferator-activated receptor-gamma coactivator-1alpha (PGC-1α) pathway patterns that may distinguish ALS from frontotemporal dementia (FTD) within an aging context. The result is a falsifiable, biomarker-oriented hypothesis model for future studies, not an evidence-based diagnostic or therapeutic algorithm.

## Introduction and background

Amyotrophic lateral sclerosis (ALS) is one of the most variable disorders in clinical neurology. Patients differ in age at onset, first affected body region, rate of decline, relative burden of upper and lower motor neuron signs, and the presence or absence of cognitive, behavioral, or mood symptoms [1,2]. Some develop bulbar or respiratory failure early. Others begin with limb weakness and maintain respiratory function for years. This diversity has made both trial design and individualized care difficult, because the diagnostic label “ALS” often groups together patients whose underlying biology may not be the same.

Current clinical staging relies heavily on site of onset, the revised ALS Functional Rating Scale (ALSFRS-R), and measures such as forced vital capacity (FVC) or clinical spread [3]. These tools are useful and have supported important natural-history research. Still, they are mostly descriptive. They do not directly represent the molecular, cellular, or circuit-level processes that may shape symptoms in each patient. Two people with the same ALSFRS-R score and the same onset site may still follow very different clinical paths, and treatment response remains hard to predict [4].

The current disease-modifying treatment context also remains limited, although it has changed in recent years. Contemporary ALS management includes riluzole and edaravone for selected patients, while tofersen is a genotype-directed antisense oligonucleotide for SOD1-ALS and was approved through an accelerated pathway supported by a reduction in plasma neurofilament light chain (NfL). Sodium phenylbutyrate/taurursodiol, also known as AMX0035 or Relyvrio, was voluntarily withdrawn from the United States and Canadian markets after the negative PHOENIX phase 3 study. This context strengthens the need for biomarker-stratified research, but it also means that new frameworks should not be interpreted as clinically actionable treatment algorithms [5-10].

Research over the last two decades has also made clear that ALS is not confined to the motor system. Cognitive and behavioral changes occur in a substantial minority of patients, and up to 15% meet criteria for frontotemporal dementia (FTD) [11,12]. Mood symptoms, including depression, are also common, with estimates ranging from 10% to 34% depending on disease stage and the method used to assess depression [13,14]. These findings support the concept of an ALS-FTD spectrum, now reflected in revised diagnostic criteria [15]. Imaging and neuropathological work further show that transactive response DNA binding protein 43 kDa (TDP-43) pathology and network disruption extend into prefrontal, limbic, and brainstem regions, not only into motor pathways [16,17].

Despite this progress, a single multi-scale framework connecting cellular mechanisms to symptom-level expression remains underdeveloped. Several biological processes are already well supported in the literature. These include microglial synaptic pruning [18], mitochondrial and energy-metabolism dysfunction [19,20], NAD+ depletion and impaired redox buffering [21,22], autophagy and proteostasis failure [23,24], and aging-related narrowing of compensatory reserve [25]. The challenge is that these mechanisms are often studied separately. A clinical framework needs to ask how they interact and why some circuits fail earlier than others. Direct experimental evidence integrating pruning, mitochondrial dysfunction, NAD+ biology, and autophagy failure into one unified ALS progression model remains limited.

This review addresses this gap by synthesizing four studies by Cheung published in 2026 [26-29] as a primary hypothesis-generating corpus. These papers connect synaptic pruning, bioenergetic stress, NAD+ dynamics, autophagy regulation, and aging-related pathway architecture into a symptom-level model of ALS. The goal is to provide a coherent scaffold that can be tested in biomarker studies, cellular systems, animal models, and future clinical trials, rather than a finished diagnostic or treatment algorithm. The present review, therefore, treats this integration as a hypothesis to be tested rather than as an established mechanism.

Methodology

Literature was selected through targeted searches of PubMed, Google Scholar, CrossRef, publisher pages, and citation tracking of landmark ALS reviews and mechanistic papers available up to May 2026. Search concepts included “amyotrophic lateral sclerosis,” “ALSFRS-R,” “frontotemporal dementia,” “ALS-FTD,” “neurofilament light chain,” “microglia,” “synaptic pruning,” “complement,” “oxidative phosphorylation,” “NAD+,” “autophagy,” “proteostasis,” “TBK1,” “C9orf72,” “PI3K-AKT-mTOR,” “PGC-1α,” “SIRT1,” “transcriptome-wide association study,” “S-PrediXcan,” “edaravone,” “tofersen,” “Relyvrio,” “sulforaphane,” and “methylmercury ALS-like pathology.”

Studies were included when they contributed to one of five evidentiary layers: clinical ALS heterogeneity and staging, ALS-FTD and extra-motor involvement, mechanistic ALS biology, genetic or transcriptomic association, or preclinical and computational hypothesis generation. Foundational non-ALS neurobiology studies were included only when they supplied a mechanism relevant to pruning, NAD+ biology, autophagy, mitochondrial reserve, or plasticity. Preprints and author-derived works were retained only as hypothesis-generating sources and are labelled as such. No formal risk-of-bias tool was applied, and no pooled effect estimates were calculated. Evidence was weighted in the following order: replicated clinical or biomarker evidence, human genetic evidence, ALS-specific preclinical evidence, general neurobiology evidence, computational modeling, and author-derived hypotheses.

## Review

Primary hypothesis-generating studies

**Figure 1:**
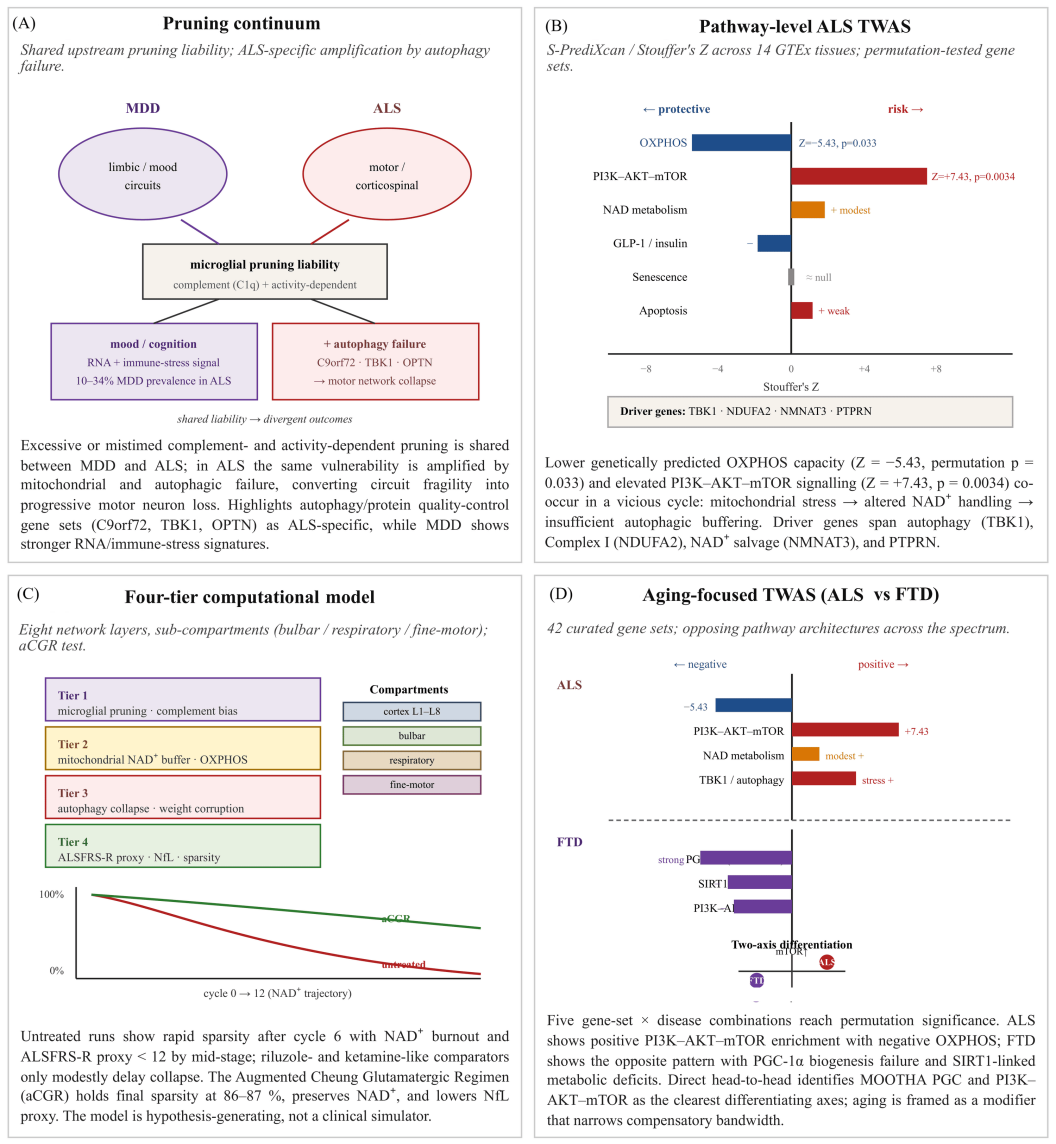
Summary of the four studies by Cheung on ALS as a primary hypothesis-generating corpus These studies are shown by evidence type rather than by implied evidentiary strength. (A) This is a peer-reviewed conceptual scaffold linking microglial pruning to ALS via shared upstream liability with major depressive disorder [26]. (B-D) These are preprints [27-29]. A common-variant pathway-level TWAS identifying OXPHOS, PI3K-AKT-mTOR, NAD metabolism, and autophagy-related axes; a temporally explicit, eight-layer four-tier simulation with bulbar, respiratory, and fine-motor sub-compartments, and an aging-focused, 42-gene-set TWAS positioning ALS and FTD as divergent pathway architectures. These works are useful for hypothesis generation but do not provide independent clinical validation. ALS: amyotrophic lateral sclerosis; TWAS: transcriptome-wide association study; OXPHOS: oxidative phosphorylation; PI3K: phosphoinositide 3-kinase; mTOR: mechanistic target of rapamycin; NAD: nicotinamide adenine dinucleotide; FTD: frontotemporal dementia Image Credit: Ngo Cheung; created using Microsoft PowerPoint (Microsoft Corporation, Redmond, Washington, United States)

In April 2026, Cheung published four connected papers that together propose a multi-scale account of ALS [26-29]; of these, three are as yet pre-prints that have not undergone peer review [27-29]. Each of these studies focuses on different levels of analysis, but the four have several assumptions in common and form a part of a broader pruning-to-autophagy continuum. None of the four studies should be treated as independent validation of the others. Because the work is recent and still requires independent validation, it is treated here as hypothesis-generating (Figure 1).

The first study proposes that synaptic plasticity fragility may form a microglial pruning continuum shared by major depressive disorder (MDD) and ALS [26]. It argues that excessive or mistimed complement- and activity-dependent pruning by microglia may remove functional synapses in both disorders. In MDD, this process is proposed to affect mood and cognitive circuits. In ALS, the same upstream vulnerability is proposed to become more destructive because it is combined with mitochondrial and autophagic failure. In this view, pruning vulnerability is converted into progressive motor network collapse when downstream repair and clearance systems cannot keep up. The study highlights autophagy and protein quality-control gene sets as especially relevant in ALS, including C9orf72, TBK1, and OPTN, while contrasting this pattern with stronger RNA and immune-stress signatures in MDD. The reported 10-34% depression prevalence in ALS is used to raise the possibility that some mood symptoms may reflect limbic-circuit pruning vulnerability rather than only psychological reaction to diagnosis or disability. This interpretation remains speculative; depression in ALS may also arise from psychosocial distress, disability, respiratory symptoms, sleep disruption, medication effects, and other non-pruning mechanisms.

The second work is a pathway-level TWAS in ALS [27]. It uses summary-based PrediXcan (S-PrediXcan) models from 14 Genotype-Tissue Expression (GTEx) v8 tissues, and large ALS genome-wide association summary statistics to test curated gene sets related to mitochondrial oxidative phosphorylation (OXPHOS), NAD metabolism, PI3K/AKT/mTOR signaling, apoptosis, glucagon-like peptide-1 (GLP-1) and insulin secretion, and senescence. The strongest protective signal is reported for OXPHOS capacity, specifically the HALLMARK_OXIDATIVE_PHOSPHORYLATION set, with Stouffer’s Z = -5.43 and permutation p = 0.033. By contrast, higher genetically predicted activity in the PI3K-AKT-mTOR signaling set is associated with greater ALS risk, with Z = +7.43 and p = 0.0034. NAD metabolism shows modest positive enrichment, while GLP-1 and insulin secretion pathways show negative enrichment, suggesting a possible protective metabolic direction. Driver genes emerging from these analyses include *TBK1*, *NDUFA2*, *NMNAT3*, and *PTPRN*. Cheung interprets these findings as consistent with a cycle in which lower OXPHOS capacity contributes to mitochondrial stress and altered NAD handling, while insufficient autophagic buffering related to TBK1 and mTOR signaling may help sustain injury. The present review does not independently reprocess those GWAS or TWAS data, audit the gene-set definitions, or test colocalization.

The third study by Cheung presents a four-tier computational model of the proposed cascade [28]. The model includes eight circuit layers and sub-compartment branching for bulbar, respiratory, and fine-motor domains. Tier 1 represents microglial pruning through complement bias and activity dependence. Tier 2 represents mitochondrial compensation and depletion using a per-layer NAD+ buffer. Tier 3 models autophagy collapse through dynamic autophagy efficiency and weight corruption. Tier 4 generates clinical-style outputs, including an ALSFRS-R proxy and an NfL proxy. In untreated simulations, sparsity accumulates rapidly after cycle 6, NAD+ levels decline, and the ALSFRS-R proxy falls below 12 by mid-stage. Riluzole-like and ketamine-like interventions delay deterioration modestly, but they do not prevent later NAD+ burnout or near-total sparsity. Early-pulsed and maintenance schedules of a multi-target glutamatergic/NAD+/autophagy regimen, termed the Augmented Cheung Glutamatergic Regimen (aCGR) in the source model, are associated with lower final sparsity, reported at 86-87%, lower NfL proxy values, and better preserved NAD+ levels. The model is presented as a tool for generating predictions, not as a validated clinical simulator.

The fourth study is an aging-focused TWAS comparing ALS and FTD [29]. It uses S-PrediXcan across 42 curated gene sets covering NAD metabolism, mitochondrial biogenesis, mTOR and AMPK signaling, senescence, apoptosis, synaptic function, and glial biology. Five gene-set-by-disease combinations reach permutation significance. In ALS, PI3K-AKT-mTOR signaling shows positive enrichment, while OXPHOS shows negative enrichment. In FTD, the strongest negative signals involve PGC-1α-related mitochondrial biogenesis, PI3K-AKT-mTOR, and a SIRT1-linked metabolic set. Direct comparison between ALS and FTD identifies MOOTHA PGC and PI3K-AKT-mTOR as the clearest differentiating pathways, with opposite directions of genetically predicted expression. Cheung proposes a two-axis model in which ALS is marked by TBK1-centered mTOR/autophagy stress and mitochondrial energy deficit, while FTD is marked by failure of PGC-1α-driven mitochondrial biogenesis and RIPK1/MAPK stress signaling. Aging is framed as a modifier that narrows the compensatory bandwidth available to motor and extra-motor networks.

**Figure 2:**
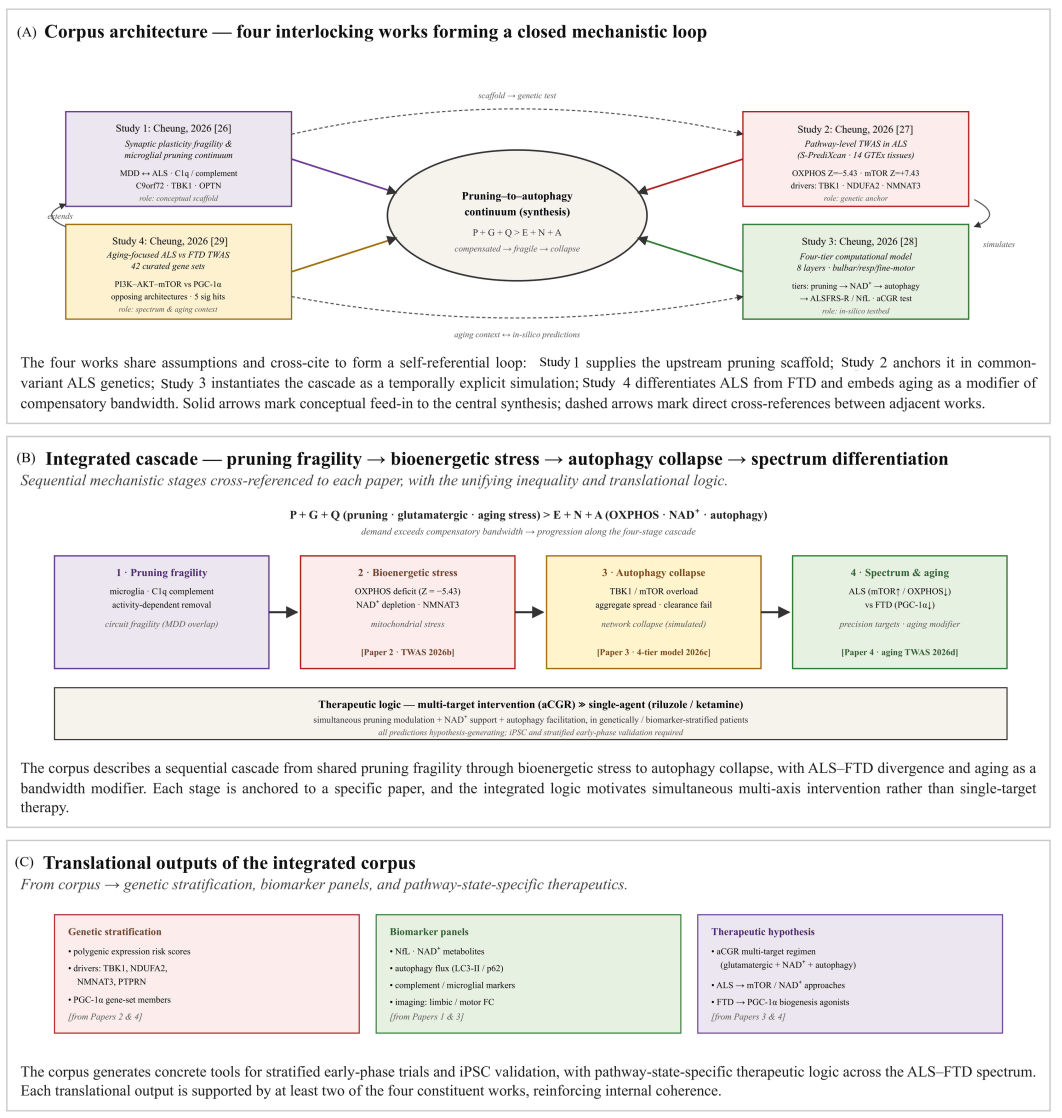
Connections between the four studies by Cheung The four studies by Cheung [26-29] are structurally interdependent and are therefore best read as a hypothesis-generating chain rather than as mutually independent evidence. The conceptual scaffold, common-variant genetic anchor, in-silico testbed, and ALS-FTD spectrum context jointly motivate the inequality P + G + Q > E + N + A. Their main value is to produce testable predictions around polygenic stratification, biomarker panels, NfL trajectories, NAD+ metabolites, autophagy flux, and pathway-state-specific interventions. Their main limitation is that the same author-derived framework supplies much of the internal support. Independent iPSC-derived motor neuron systems, animal models, longitudinal biomarker cohorts, and externally replicated TWAS analyses are required before clinical interpretation is justified. ALS: amyotrophic lateral sclerosis; FTD: frontotemporal dementia; NAD+: oxidized nicotinamide adenine dinucleotide; NfL: neurofilament light chain; iPSC: induced pluripotent stem cells; TWAS: transcriptome-wide association study Image Credit: Ngo Cheung; created using Microsoft PowerPoint (Microsoft Corporation, Redmond, Washington, United States)

Taken together, the four works form a closed mechanistic loop (Figure 2). The framework is internally consistent, but each element remains preliminary and needs independent testing. Internal consistency should not be confused with independent corroboration. The risk of circular hypothesis reinforcement is real because the four works are author-derived and mechanistically interdependent.

Table 1 explains the evidentiary hierarchy. The purpose is not to dismiss author-derived work, but to prevent associative, preclinical, computational, and speculative claims from being treated as equivalent to established clinical evidence.

**Table 1:** Evidence status of the main components used in the framework TWAS: transcriptome-wide association study; ALS: amyotrophic lateral sclerosis; ALSFRS-R: revised ALS Functional Rating Scale; FVC: forced vital capacity; FTD: frontotemporal dementia; OXPHOS: oxidative phosphorylation; NAD+: oxidised nicotinamide adenine dinucleotide

Domain	Main evidence type	How it is used here	Status and limitation
ALS heterogeneity, ALSFRS-R, FVC, clinical staging	Clinical cohorts, outcome-scale literature, management guidelines	Justifies symptom-domain rather than purely diagnosis-level framing	Established clinical context; not a molecular mechanism by itself
Current disease-modifying therapy context	Clinical trials, guidelines, regulatory records	Shows why better stratification remains needed despite riluzole, edaravone, and genotype-directed tofersen	Clinically relevant, but does not validate the present model; AMX0035 withdrawal also cautions against premature translational claims
ALS-FTD and extra-motor involvement	Clinical, imaging, and neuropathological evidence	Supports prefrontal, limbic, and brainstem circuit mapping	Well supported at syndrome level; mechanism of each symptom remains heterogeneous
Microglial pruning and complement	Developmental/general neurobiology plus ALS-specific complement observations	Supports pruning pressure P as a plausible circuit stressor	Complement-mediated pruning is not yet proven as a unified ALS progression driver; schizophrenia C4 data are analogical, not ALS-specific
Mitochondrial/OXPHOS reserve	ALS tissue, muscle, metabolic, and preclinical literature	Supports energy reserve E in the equation	Biologically plausible and widely studied; patient-level thresholds remain unvalidated
NAD+ biology	Aging biology, neurodegeneration literature, limited ALS-specific preclinical/human data	Supports NAD+ reserve N as a compensatory buffer	ALS-specific clinical evidence is limited; NAD+ should not be treated as a proven central driver
Autophagy/proteostasis	Genetic and functional ALS evidence involving TBK1, OPTN, C9orf72, and related pathways	Supports autophagic reserve A and collapse state	Strong mechanistic plausibility; timing and measurable clinical thresholds remain unresolved
Nrf2/SIRT1 and sulforaphane-related multitarget pharmacology	Recent methylmercury-induced ALS-like rat model and broader preclinical neuroprotection literature	Provides external preclinical plausibility for antioxidant, anti-inflammatory, myelin, neurofilament, and systemic-reserve axes	Not a human ALS trial and not direct validation of this framework
Cheung 2026 corpus [26-27]	One peer-reviewed hypothesis paper and three Figshare preprints with TWAS and computational analyses	Provides the immediate scaffold for the proposed integrated model	Author-derived, not independently validated, and at risk of circular reinforcement
Three-state model and inequality	Present synthesis	Provides a proposed research language for compensated plasticity, fragile plasticity, and network collapse	Theoretical; requires prospective biomarker operationalization and falsification

The integrated symptom-level precision neurology framework

The framework developed treats ALS symptoms as the visible result of circuit-level decompensation. Symptoms are not interpreted simply as direct consequences of motor neuron death. Instead, each symptom domain is understood as the point at which a specific neural circuit can no longer preserve plasticity, connectivity, and function under the combined pressure of pruning, energy demand, and proteostatic stress.

Core Premise

The central premise is that ALS symptoms emerge when region-specific motor and extra-motor circuits lose the energy and clearance capacity needed to maintain normal function. Microglial pruning may reduce synaptic reserve. Mitochondrial OXPHOS and NAD+ buffering determine whether compensation can continue. Autophagy and proteostasis determine whether damaged proteins, organelles, and circuit elements can be cleared before they become toxic. Aging and senescence progressively narrow the remaining adaptive capacity. Under this model, progression is not a simple uniform decline. It is a set of transitions across biological states, and the timing of those transitions differs by circuit and by patient. This differs from a purely cell-loss model by treating symptoms as threshold events in circuit reserve. Motor neuron degeneration remains central, but it is not the only level at which progression is represented.

Symptom-to-Circuit Mapping

Different ALS symptom clusters can be mapped to different vulnerable circuit compartments. Limb weakness is interpreted as failure within corticospinal and spinal motor networks, where motor synapse pruning interacts with mitochondrial energy deficit. Bulbar dysfunction is linked to brainstem and bulbar motor compartments, which face high functional demand and may be especially vulnerable when pruning and autophagy stress occur together. Respiratory decline is usually later and is associated with energetic and autophagic collapse in phrenic and respiratory motor networks. Fine-motor loss reflects early synaptic sparsity and impaired plasticity in high-precision corticospinal circuits. Mood and cognitive symptoms are readouts of limbic and prefrontal vulnerability, possibly involving microglial pruning liability and immune or RNA-related stress. Fatigue and exertional intolerance are interpreted as global signs of reduced metabolic reserve when OXPHOS and NAD+ compensation reach their limits. This mapping is not meant to be rigid. It is a way to organize clinical observation around plausible biological mechanisms. It is not yet a validated symptom-localization system.

Energy-Constrained Plasticity

A key organizing idea is that plasticity has a cost. Synaptic remodeling, compensatory sprouting, ongoing neurotransmission, protein turnover, mitochondrial repair, and microglial surveillance all require energy and stress-buffering capacity. When those resources are sufficient, pruning and remodeling may be adaptive. When they are limited, the same processes can become damaging. In circuits that are already under high demand, pruning pressure combined with weak bioenergetic or autophagic reserve may accelerate synaptic sparsity rather than support repair. This perspective links synaptic biology with metabolic and proteostatic research and may help explain why some patients decline quickly while others maintain function longer despite similar levels of motor system involvement.

Three-State Biological Symptom Model

The model separates circuit states into compensated plasticity, fragile plasticity, and network collapse. These states are intended as broad biological categories, not fixed clinical stages. They are proposed research states rather than empirically validated ALS subtypes.

In compensated plasticity, pruning pressure may already be present, but OXPHOS remains adequate, NAD+ buffering is preserved, and autophagy continues to function. Symptoms are absent, subtle, or focal. A patient may report occasional cramps, mild fine-motor inefficiency, fatigue, or fluctuating mood or cognitive changes. The circuit still has enough reserve to preserve connectivity despite stress.

In fragile plasticity, energy demand begins to exceed mitochondrial reserve. NAD+ compensation is recruited but becomes strained. Microglial pruning erodes synaptic redundancy, and autophagy is stressed but not fully collapsed. Clinically, this stage may correspond to clearer limb, bulbar, or fine-motor decline, increased fatigability, and the appearance or worsening of mood or cognitive symptoms. The ALSFRS-R slope may begin to steepen, but some compartments may still retain meaningful reserve.

In network collapse, NAD+ buffering fails, autophagy and proteostasis break down, and synaptic sparsity becomes self-reinforcing. NfL-like injury signals rise, and respiratory or bulbar circuits may deteriorate quickly. Functional decline is steep, and compensatory capacity is limited. These states are not assumed to move together across all circuits. A single patient may have fragile plasticity in one compartment while another remains compensated. This asynchrony may account for the patchy and uneven progression often seen in ALS.

Operationally, compensated plasticity would have to be defined in future studies by stable ALSFRS-R subdomain slopes over a prespecified interval, low or stable NfL, preserved respiratory measures, and no clear deterioration in candidate OXPHOS, NAD+, or autophagy markers. Fragile plasticity would require emerging subdomain decline with rising but not sharply accelerating NfL, evidence of metabolic or NAD+ stress, and no decisive autophagy-collapse signature. Network collapse would require steep subdomain decline, respiratory or bulbar acceleration, milestone events such as non-invasive ventilation or gastrostomy, rapidly rising or high NfL, and convergent evidence of impaired energy, NAD+, and autophagic reserve. These are candidate research definitions, not clinical thresholds.

The Compact Mechanistic Equation

The core dynamic can be summarized as follows: Sᵢ(t) = f(Pᵢ(t), Eᵢ(t), Nᵢ(t), Aᵢ(t), Gᵢ(t), Qᵢ(t)). In this expression, Sᵢ(t) is the symptom burden in circuit domain i at time t. Pᵢ is pruning pressure, Eᵢ is mitochondrial OXPHOS capacity, Nᵢ is NAD+ compensation and reserve, Aᵢ is autophagy and proteostasis capacity, Gᵢ is glutamatergic and excitability burden, and Qᵢ is the aging, senescence, and inflammatory context. Symptoms are expected to appear or worsen when pruning pressure, glutamatergic load, and aging-related stress exceed the circuit’s combined energy, NAD+, and autophagic reserve: Pᵢ + Gᵢ + Qᵢ > Eᵢ + Nᵢ + Aᵢ.

This formulation is intentionally simple. It does not define exact mathematical interaction terms. Its purpose is to provide a common language for why different circuits fail at different times and why multi-target approaches may be more useful than interventions aimed at only one mechanism. The variables should not be treated as directly measurable constants until standardized biomarker definitions are developed. Candidate operational measures are shown in Table 2.

**Table 2:** Candidate operationalization of the framework variables for future studies

Variable	Candidate measures	Example validation endpoint
Sᵢ: symptom burden in circuit domain i	ALSFRS-R total and subdomain scores, bulbar scales, hand-function measures, FVC or slow vital capacity, cognitive screening, depression or anxiety scales, time to ventilation, gastrostomy, or death	Whether biomarker-defined states predict symptom-domain slope better than baseline ALSFRS-R alone
Pᵢ: pruning pressure	Complement markers such as C1q, C3, C4; HLA/MHC-related expression; microglial activation imaging where available; synaptic-density imaging where available; CSF or plasma complement panels	Association with regional connectivity loss, cognitive or mood symptoms, and motor spread
Eᵢ: OXPHOS capacity	OXPHOS transcriptomic signatures, lactate/pyruvate ratio, acylcarnitines, muscle or induced pluripotent stem cell-derived motor neuron respirometry, phosphorus magnetic resonance spectroscopy in research settings	Prediction of ALSFRS-R motor or respiratory subdomain decline
Nᵢ: NAD+ compensation and reserve	NAD+/NADH, NADP+/NADPH, nicotinamide and related metabolites, NMNAT and sirtuin expression, redox markers	Prediction of transition from stable or slowly progressive disease to rising NfL and steeper functional decline
Aᵢ: autophagy and proteostasis capacity	LC3-II, p62/SQSTM1, LAMP1/2, lysosomal markers, TBK1/OPTN/C9orf72 genotype or expression, TDP-43-related assays where available	Association with NfL acceleration, motor-unit loss, or rapid clinical milestones
Gᵢ: glutamatergic and excitability burden	Transcranial magnetic stimulation indices of cortical hyperexcitability, electromyography measures, fasciculation burden, magnetic resonance spectroscopy glutamate measures, excitotoxicity assays in cellular systems	Association with cramps, spasticity, early spread, and response to glutamate-modulating interventions
Qᵢ: aging, senescence, and inflammatory context	Chronological age, frailty, inflammatory cytokines, p16INK4a-related measures, senescence-associated secretory phenotype markers, mTOR/PGC-1α/SIRT1 signatures	Modification of reserve, progression rate, and response to biologically matched interventions

NAD+ as the Central Compensatory Buffer

Within this framework, NAD+ is treated as a central compensatory buffer during the shift from compensated to fragile plasticity. Early in the process, NAD+ reserve supports mitochondrial function, redox balance, and repair. This allows circuits to tolerate pruning and increased energy demand. As stress accumulates, NAD+ is increasingly consumed, and compensation becomes more costly. Once the buffer is exhausted, autophagy efficiency falls and network sparsity accelerates. Phase-dependent NAD+ dynamics may therefore help explain the clinical shift from subtle or fluctuating symptoms to faster and more persistent decline. This emphasis is mechanistically plausible but not yet clinically proven in ALS. NAD+ is best viewed as one candidate buffer within a broader reserve system rather than as an established master regulator of ALS progression.

Aging and Senescence as Narrowing of Adaptive Bandwidth

Aging is not treated as a separate disease process in this model. It is treated as a gradual narrowing of adaptive bandwidth. With age, OXPHOS and NAD+ reserve decline, while mTOR-linked stress and autophagy impairment may rise. Plasticity becomes more fragile, and pruning becomes less reversible. The framework also incorporates pathway divergence between ALS and FTD. In ALS, genetically predicted PI3K-AKT-mTOR activity tends to be elevated while OXPHOS is reduced. This pattern is consistent with TBK1-centered mTOR and autophagy stress layered on mitochondrial energy deficit. In FTD, the strongest signals involve failure of PGC-1α-driven mitochondrial biogenesis, along with negative enrichment of PI3K-AKT-mTOR and SIRT1-linked metabolic sets. These opposite pathway patterns suggest that aging may amplify different vulnerabilities across the ALS-FTD spectrum and may have implications for therapeutic targeting. At present, this remains an associative TWAS-derived hypothesis rather than a validated disease-classification scheme.

Multi-Axis Patient Stratification

**Figure 3:**
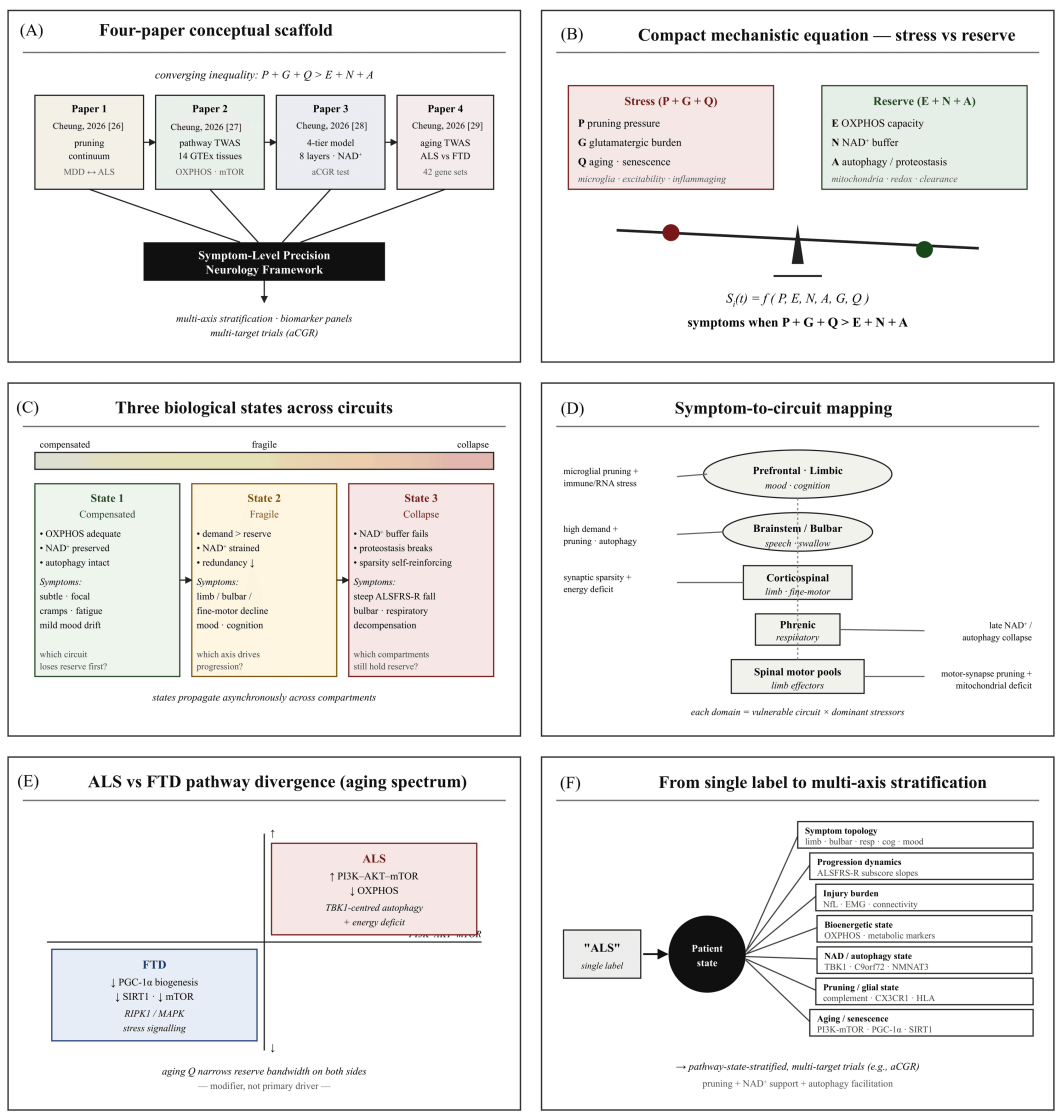
Shared background framework for symptom-level precision neurology in ALS This figure should be read as a proposed testing framework. The four studies by Cheung [26-29] provide a hypothesis scaffold linking synaptic pruning/autophagy vulnerability, TWAS-derived genetic axes, temporal compartmental dynamics, and aging-related loss of reserve. Independent evidence supports several individual domains, including ALS heterogeneity, extra-motor involvement, mitochondrial stress, autophagy genetics, NfL, and treatment limitations. The central inequality, P + G + Q > E + N + A, remains a compact hypothesis rather than a calibrated mathematical model. The figure is intended to guide biomarker-state validation, not current clinical decision-making. TWAS: transcriptome-wide association study; NfL: neurofilament light chain; ALS: amyotrophic lateral sclerosis Image Credit: Ngo Cheung; created using Microsoft PowerPoint (Microsoft Corporation, redmond, Washington, United States)

The model supports a shift away from a single disease label and toward multi-axis stratification. Relevant axes include symptom topology, such as limb onset, bulbar onset, respiratory involvement, fine-motor decline, and cognitive or mood features. Progression dynamics would include ALSFRS-R total and subdomain slopes, as well as respiratory and bulbar trajectories. Injury burden could include NfL, electromyography (EMG) progression, and connectivity measures when available. Bioenergetic state could be assessed through OXPHOS-related signatures and metabolic markers. NAD and autophagy state could include NAD-pathway genes, TBK1, C9orf72, and other autophagy-related signatures. Pruning and glial state could be represented by complement, human leukocyte antigen (HLA)/major histocompatibility complex (MHC), CX3CR1, and microglial markers. Aging or senescence context could include PI3K-AKT-mTOR, PGC-1α, and SIRT1-linked signatures. Such stratification could help refine natural history studies, improve trial enrichment, and guide future precision approaches (Figure 3).

Subtype-specific handling would be essential. SOD1-ALS should remain anchored to genotype-directed evidence and tofersen eligibility where appropriate. C9orf72 ALS/FTD would require explicit cognitive, behavioral, RNA-processing, and autophagy-related endpoints. Bulbar-onset ALS should be analyzed with speech, swallowing, nutrition, and respiratory trajectories rather than total ALSFRS-R alone. Respiratory-onset ALS would need early respiratory milestones and ventilation endpoints. Lower-motor-neuron-predominant phenotypes may require EMG, motor-unit, neuromuscular-junction, and peripheral axonal measures. The framework should therefore be tested within ALS subtypes, not only across pooled ALS cohorts.

Grounding in established peer-reviewed literature

The proposed framework draws on established clinical, cellular, molecular, imaging, and genetic findings in ALS and related disorders.

Current ALS Therapeutic Context

Disease modification in ALS remains limited. Riluzole has long-standing randomized-trial support, edaravone has evidence in a selected ALS trial population, and tofersen represents a genotype-directed approach for SOD1-ALS with regulatory approval linked to plasma NfL reduction. The withdrawal of AMX0035/Relyvrio after negative phase 3 data is a reminder that promising biological rationale and early clinical signals can fail in confirmatory testing. This treatment landscape supports the need for better stratification while also arguing for caution in moving from hypothesis to intervention [5-10].

Clinical Heterogeneity, ALSFRS-R, and Progression Variability

Longitudinal studies show wide variation in ALS onset site, progression rate, and respiratory involvement [1,2,4]. The ALSFRS-R, which includes respiratory subscores, remains the most widely used clinical outcome measure and has supported detailed natural-history work [3]. These observations support a symptom-domain approach rather than reliance only on global staging.

Cognitive, Mood, and Psychiatric Manifestations

Cognitive and behavioral symptoms occur across ALS stages, and some patients meet criteria for FTD [11,12,30]. Depression is reported in 10-34% of patients, depending on stage and assessment method [13,14,31]. Revised criteria now explicitly recognize the ALS-FTD spectrum [15]. These findings are consistent with the idea that limbic and prefrontal circuits may be affected in ALS, but they do not by themselves prove a pruning-mediated mechanism. Psychological reaction to diagnosis, disability, sleep disturbance, respiratory compromise, caregiver stress, and medication effects remain important alternative or coexisting explanations for depression in ALS.

Microglial Pruning, Complement Cascade, and Synaptic Vulnerability

Microglia participate in developmental and activity-dependent synaptic pruning through complement tagging [18,32,33]. Complement component 4 (C4) variation has been linked to synaptic elimination risk in schizophrenia, and microglial surveillance of synaptic function has been demonstrated in vivo [34,35]. These studies are relevant because they show that complement-mediated synaptic selection can shape human-relevant neural circuits, but they are not direct evidence of ALS-specific pruning. ALS-specific relevance is supported more cautiously by observations such as complement activation at motor end-plates and broader inflammatory-complement hypotheses in motor neuron disease [36]. These mechanisms provide biological plausibility for a pruning-continuum model that includes both ALS and MDD, but the extrapolation remains incomplete without ALS-specific functional validation.

Early Synaptic/Neuromuscular Junction (NMJ) Dysfunction and Distal Axonopathy

Human and animal studies suggest that ALS can involve early distal axonopathy and neuromuscular junction instability before overt motor neuron loss [37,38]. Non-neuronal cells also contribute to ALS pathobiology [39]. Selective vulnerability and pruning of phasic motoneuron axons have also been reported [40]. These findings are consistent with the model’s emphasis on early synaptic sparsity and circuit vulnerability, although they do not prove that microglial pruning is the initiating event.

Mitochondrial Dysfunction, Oxidative Phosphorylation, and Metabolic Reserve

Mitochondrial alterations are well documented in ALS spinal cord and muscle, and energy-metabolism deficits have been proposed as core features of the disease [19,41,42]. Broader reviews describe energy metabolism as an underappreciated therapeutic opportunity in ALS [20]. These observations support the bioenergetic-reserve component of the framework.

NAD+ Biology, Redox Buffering, and Aging-Related Compensation Failure

NAD+ decline is a recognized feature of aging and neurodegeneration. It contributes to pseudohypoxic signaling and impaired nuclear-mitochondrial communication [21,22]. NAD+ replenishment has been shown to improve mitophagy and healthspan in model systems [43,44]. Reviews of NAD+ biology in brain aging and neurodegeneration further support its role in stress resistance and metabolic compensation [45,46]. ALS-specific evidence is narrower, but work examining the NAD+ biosynthetic pathway in ALS patients and hSOD1-linked mouse models provides some disease-relevant support for studying NAD+ modulation in ALS biology [47]. These findings underpin the role assigned here to NAD+ buffering, while leaving its clinical weight unresolved.

Autophagy/Proteostasis Collapse and Key ALS Genes

Several ALS-associated genes, such as *TBK1,* *OPTN*, and *C9orf72*, converge on autophagy and proteostasis. TBK1 haploinsufficiency causes familial ALS and FTD [24]. OPTN mutations were identified as ALS-associated mutations [48]. C9orf72 expansions impair autophagy initiation [49,50]. Large exome-sequencing studies have also highlighted autophagy-related pathways in ALS risk [23]. These genetic findings support the idea that autophagy failure can amplify ALS-specific progression, but they do not establish the timing of autophagy collapse in individual patients.

PI3K-AKT-mTOR Signaling in Plasticity and Neurodegeneration

mTOR signaling is involved in plasticity, memory, and disease [51,52]. The PI3K/AKT/mTOR pathway regulates autophagy and the clearance of protein aggregates in neurodegeneration [53]. mTOR inhibition has also been discussed as a therapeutic strategy in neurodegenerative disease [54]. These mechanisms are consistent with the TWAS finding of risk-associated PI3K-AKT-mTOR enrichment in ALS, but that enrichment remains associative and should not be taken as proof that broad mTOR inhibition would be beneficial in ALS.

Nrf2/SIRT1 and Recent Sulforaphane Preclinical Evidence

A recent molecular neurobiology study by Mukherjee et al. tested sulforaphane in methylmercury-induced ALS-like pathology in rats, with comparison to omaveloxolone and dimethyl fumarate [55]. In their preclinical toxin model, sulforaphane, particularly at 4 mg/kg, was reported to upregulate Nrf2, HO-1, and SIRT1 while suppressing IL-1β, TNF-α, Bax, caspase-3, p75NTR, PI3K/Akt, and MAPK signaling. The reported effects included improvement in grip strength, locomotor performance, spatial memory, depressive-like behavior, demyelination, neuronal architecture, hepatic enzymes, skeletal muscle integrity, redox balance, neurofilament and myelin-associated proteins, and hematological alterations. Their study is relevant because it independently touched several axes considered in the present framework: oxidative stress, inflammatory signaling, survival pathways, myelin and neurofilament integrity, and systemic reserve. However, it remains a methylmercury-induced ALS-like rat model rather than a genetic or sporadic human ALS study. It should therefore be interpreted as external preclinical plausibility, not as validation of the three-state model or of any clinical regimen.

PGC-1α, Mitochondrial Biogenesis, and SIRT1 in ALS-FTD Energy Biology

PGC-1α is a key regulator of mitochondrial biogenesis and energy metabolism [56,57]. SIRT1 activates PGC-1α, and altered sirtuin expression has been reported in ALS [58]. Work on mitophagy and mitochondrial biogenesis during aging further links these pathways to reserve capacity [59]. The opposing PGC-1α-related signals described between ALS and FTD are consistent with this broader literature, but their disease-specific interpretation requires replication and functional validation.

Cellular Senescence, Inflammaging, and Narrowed Reserve

Cellular senescence and inflammaging are hallmarks of aging and contribute to tissue dysfunction [25,60-62]. p16Ink4a-positive senescent cells have been shown to shorten healthy lifespan [63]. These processes support the view that aging narrows the compensatory bandwidth available to vulnerable neural circuits.

NfL as an Injury Biomarker

NfL in blood and cerebrospinal fluid has emerged as a sensitive marker of neuronal injury and has prognostic value in ALS [64-67]. Its use as an injury proxy in computational models is therefore grounded in clinical biomarker research. However, NfL is an injury marker rather than a mechanism-specific readout; it cannot by itself distinguish pruning, mitochondrial injury, autophagy failure, or excitotoxicity.

Glutamatergic Excitability, Riluzole, and Ketamine-Like Mechanisms

Glutamate excitotoxicity remains an important ALS hypothesis [68]. Riluzole modulates glutamatergic transmission and remains part of ALS treatment practice [6,69,70]. Ketamine and its metabolites have been linked to mTOR-dependent synapse formation and rapid antidepressant effects [71-73]. These findings support the inclusion of glutamatergic burden in the model. The ketamine-related references are included for synaptogenic and mTOR-linked plasticity mechanisms, not as evidence that ketamine treats ALS or should be used for ALS disease modification.

Genetic Architecture and TWAS Methodology Foundation

Large-scale genome-wide association studies have clarified the polygenic architecture of ALS [74-76]. Transcriptome-wide association methods, including PrediXcan and S-PrediXcan, link genetic variation to tissue-specific predicted gene expression [77,78]. These methods provided the basis for the pathway-level and aging-focused TWAS analyses in the four studies by Cheung [26-29]. TWAS is useful for prioritizing genes and pathways, but it remains associative and can be confounded by linkage disequilibrium, tissue-model limitations, gene-set curation, and lack of colocalization.

Extra-Motor Networks and Staged Pathology

Neuroimaging and neuropathology show that ALS affects networks beyond the motor system [17,79,80]. TDP-43 pathology can spread in staged patterns involving prefrontal and limbic regions [16]. These findings support a circuit-compartment approach to symptom mapping.

Novel hypotheses and testable predictions

The framework generates several hypotheses that can be tested using existing and emerging methods. These are proposed as starting points for empirical study rather than as established conclusions. All hypotheses below are speculative until externally validated.

Primary Hypothesis

The central hypothesis is that ALS progression reflects transitions across compensated plasticity, fragile plasticity, and network collapse. These transitions occur when pruning pressure, glutamatergic load, and aging-related stress exceed a circuit’s combined OXPHOS capacity, NAD+ reserve, and autophagic clearance. This formulation is consistent with the temporal dynamics described in the four-tier computational model and with the pathway-level signals reported in the TWAS analyses. It has not yet been prospectively validated in patients.

Hypothesis 1

Early depressive or cognitive symptoms in some patients may reflect pruning-related vulnerability in limbic and prefrontal circuits, rather than arising only as a reaction to diagnosis or disability. This hypothesis follows from the pruning continuum proposed by Cheung [26] and from the observed prevalence of mood symptoms in ALS cohorts [13]. It also fits evidence that microglial pruning mechanisms are relevant across neuropsychiatric and neurodegenerative contexts [34]. A direct ALS test would require longitudinal mood and cognitive phenotyping, complement or microglial markers, neuroimaging connectivity measures, and adjustment for disability, respiratory status, sleep, and psychosocial burden.

Hypothesis 2

Patients whose circuits remain in the fragile-plasticity state while autophagy capacity is still partly preserved are expected to progress more slowly and respond better to multi-tier interventions than patients who have already entered autophagy-collapse. This distinction may help explain why some people retain function longer despite comparable motor neuron involvement, and why single-target therapies have produced only modest benefits. Candidate endpoints include ALSFRS-R subdomain slopes, NfL trajectories, respiratory measures, autophagy markers such as p62/SQSTM1 and LC3-related readouts in experimental systems, and time to ventilation or gastrostomy.

Hypothesis 3

Low genetically predicted OXPHOS capacity combined with elevated PI3K-AKT-mTOR activity may identify a subgroup at increased risk for rapid NAD+ depletion and earlier respiratory involvement. This hypothesis follows from the protective direction of OXPHOS and the risk-associated direction of PI3K-AKT-mTOR signaling reported in the pathway-level TWAS. It also suggests that polygenic expression scores based on driver genes such as NDUFA2, NMNAT3, and TBK1 could be explored for stratification. Such scores should be tested only as research tools and should be benchmarked against established predictors such as age, site of onset, baseline ALSFRS-R, FVC, genotype, diagnostic delay, and NfL.

Hypothesis 4

Opposing pathway architectures in ALS and FTD may predict different treatment responsiveness across the spectrum. In ALS, PI3K-AKT-mTOR enrichment with reduced OXPHOS may make mTOR modulation or NAD+-supporting approaches more relevant. In FTD-predominant presentations, PGC-1α biogenesis failure and negative PI3K-AKT-mTOR enrichment may point more toward PGC-1α agonists or mitochondrial biogenesis enhancers. This hypothesis is consistent with broader work on mitochondrial biogenesis, mTOR signaling, and neurodegeneration. It remains associative and should not be used to select treatment outside research.

Hypothesis 5

A multi-target regimen that addresses pruning dynamics, NAD+ support, and autophagy facilitation is predicted to delay critical sparsity thresholds more effectively than single-agent riluzole-like or ketamine-like interventions. This prediction comes from computational simulations in which the source model’s multi-target regimen preserved NAD+ levels and reduced final sparsity more than comparator arms. Testing this idea in induced pluripotent stem cell-derived motor neuron networks and animal models would be a logical next step. Clinical testing would require prior pharmacologic justification, dose rationale, safety assessment, interaction review, and target-engagement biomarkers. A simulation alone cannot support clinical use.

Specific Measurable Predictions

Several measurable predictions follow. In longitudinal cohorts, rising NfL should correlate more strongly with the transition from fragile plasticity to collapse than with overall ALSFRS-R decline alone. Imaging or connectivity measures should show earlier limbic and prefrontal disruption in patients who later develop prominent mood or cognitive symptoms. In stratified early-phase trials, patients with low OXPHOS signatures and high PI3K-AKT-mTOR activity should be more likely to show target engagement from NAD+-supporting or autophagy-modulating agents. In iPSC models, lines carrying TBK1 or C9orf72 variants should show faster NAD+ depletion and autophagy impairment under pruning-mimetic stress than control lines. Failure of these predictions would argue against the framework or require substantial revision.

Falsification and prospective validation roadmap

A useful hypothesis framework should be able to fail. The model would be weakened if pruning or complement markers do not associate with mood, cognitive, imaging, or progression phenotypes after adjustment for known clinical predictors; if NAD+ metabolites do not change across proposed state transitions; if OXPHOS and PI3K-AKT-mTOR signatures fail to predict ALSFRS-R subdomain slopes, NfL trajectories, respiratory decline, or milestone events; if autophagy markers do not distinguish fragile plasticity from network collapse; or if iPSC-derived motor neuron and microglia co-culture systems do not reproduce the predicted interaction among pruning stress, NAD+ strain, and impaired autophagy.

A prospective validation study could enroll patients with early ALS, including SOD1-ALS, C9orf72 ALS/FTD, bulbar-onset ALS, respiratory-onset ALS, and lower-motor-neuron-predominant phenotypes. Baseline testing would include ALSFRS-R subdomains, FVC or slow vital capacity, NfL, genotype, cognitive and mood measures, metabolic markers, NAD-related metabolites, inflammatory and complement markers, and autophagy/proteostasis markers where feasible. Follow-up every three to six months could test whether biomarker-defined states predict ALSFRS-R subdomain slopes, NfL trajectories, respiratory decline, time to non-invasive ventilation, time to gastrostomy, and survival. Statistical analysis would require mixed-effects models for repeated ALSFRS-R and NfL measures, time-to-event models for milestones, correction for multiple comparisons, and prespecified adjustment for age, sex, onset site, diagnostic delay, baseline ALSFRS-R, baseline respiratory function, genotype, and baseline NfL.

Implications for research, validation, and future trial design

The framework has several implications for ALS research and clinical study design. These implications are framed as a validation roadmap, not as current clinical practice.

Shift From Diagnosis-Centric to Multi-Axis, Symptom-Circuit-State Stratification

Future studies could move beyond enrolling patients only by ALS diagnosis. Participants could instead be stratified by symptom topology, progression dynamics, injury burden, bioenergetic state, NAD/autophagy signatures, pruning and glial markers, and aging context. This would allow investigators to interpret natural history and treatment effects with more biological precision. Such stratification should be judged by whether it improves prediction beyond existing clinical and biomarker models.

Proposed Biomarker Panels and Genetic/polygenic Expression Risk Scores for Patient Enrichment

Biomarker panels could combine NfL, metabolic markers, NAD-pathway gene expression, autophagy flux measures, and polygenic scores derived from TWAS driver genes. These tools could help identify patients most likely to show target engagement from specific interventions. Enrichment strategies of this kind are already used in other areas of medicine and may improve signal detection in ALS trials. However, none of these proposed panels is currently validated for patient selection in ALS.

Rationale for Early-Phase Trials of Multi-Target Regimens in Genetically or Biologically Stratified Cohorts

The computational finding that multi-tier targeting outperformed single-agent approaches supports early-phase testing of combined regimens only after preclinical validation. Such regimens might include components that modulate pruning, support NAD+ metabolism, and facilitate autophagy. Testing them in genetically or biomarker-defined early-stage cohorts could increase the chance of detecting meaningful biological and clinical effects. Before any human trial, the regimen would need a non-eponymous pharmacologic definition, dose justification, safety monitoring plan, interaction assessment, and target-engagement endpoints.

Potential for Cross-Disease Insights

The pruning continuum linking ALS and MDD, together with the opposing PI3K-AKT-mTOR and PGC-1α architectures distinguishing ALS from FTD, creates opportunities for cross-disease learning. Strategies developed for one condition may inform another, especially where microglial pruning, mitochondrial stress, or autophagy failure overlap. Cross-disease extrapolation should remain conservative unless supported by disease-specific experiments.

Broader Relevance to Other Neurodegenerative and Neuropsychiatric Disorders

The broader principle is that energy-constrained plasticity may limit circuit resilience. This idea is likely relevant beyond ALS. Similar frameworks could be explored in other disorders marked by synaptic vulnerability, mitochondrial dysfunction, and proteostatic stress. Such work may reveal shared therapeutic entry points across diseases that appear clinically distinct.

Limitations

Several limitations should be stated clearly. The studies by Cheung [26-29] are recent and should be treated as hypothesis-generating. Their claims require independent validation and functional follow-up before they can be considered established. Because the framework relies heavily on author-derived works, there is a risk of circular hypothesis reinforcement. The evidence-grade table (Table 1) is intended to make that risk explicit, but it does not remove it. Independent replication by groups without intellectual investment in the model is essential. Secondly, the four-tier model simplifies complex biology into a mesoscale neural-network framework. Although it reproduces broad clinical trajectories, it cannot capture all molecular interactions, cell-type dynamics, or circuit heterogeneity present in human ALS. Thirdly, TWAS studies identify associations between genetically predicted gene expression and disease risk. They do not prove causality. The pathway-level signals reported in the studies by Cheung [26-29] are consistent with the proposed model, but they require functional testing in cellular and animal systems. Colocalization, fine-mapping, independent replication, and cell-type-specific validation would be needed before causal interpretation.

Fourthly, large longitudinal datasets that combine clinical measures, molecular markers, imaging, pruning-related markers, NAD+ metabolites, autophagy flux, and circuit connectivity are not yet available at the scale needed to test the three-state model directly. Prospective studies will be needed to evaluate the timing and order of the proposed transitions. Fifthly, the model maps symptoms to broad circuit domains, but real neural networks are highly interconnected. Treating compartments as partly separable is a useful heuristic, yet it may not fully capture the distributed nature of ALS pathology. Future versions of the framework will need to represent network-level interactions more explicitly.

Keeping these limitations in mind, it should be noted that the present framework does not replace established ALS evaluation, genetic testing where indicated, respiratory monitoring, multidisciplinary care, or evidence-based treatment. Its intended use is to generate falsifiable research questions. As such, it should not be used to make diagnostic, prognostic, or therapeutic decisions.

It should also be noted that while the recent sulforaphane study in methylmercury-induced ALS-like pathology [55] is useful because it supplies external preclinical evidence touching Nrf2, HO-1, SIRT1, inflammatory, apoptotic, myelin, neurofilament, and systemic markers, toxin-induced ALS-like pathology is not the same as sporadic ALS, SOD1-ALS, C9orf72 ALS/FTD, or other genetic forms. Its findings should not be extrapolated to human treatment without additional disease-relevant models and clinical trials.

## Conclusions

Synaptic pruning, mitochondrial and NAD+ biology, autophagy regulation, and aging-associated pathway divergence have been linked in this review into a symptom-level precision neurology framework for ALS. When placed alongside established literature on ALS heterogeneity, extra-motor involvement, pruning biology, mitochondrial stress, proteostasis, and senescence, the model generates a set of testable hypotheses while remaining provisional.

Its value will depend on whether these predictions can be supported, revised, or rejected through empirical work using iPSC-derived systems, animal models, longitudinal biomarker cohorts, neuroimaging, and early-phase clinical trials designed around biological state rather than diagnosis alone. If validated, this approach could support pathway-state-stratified trials in ALS and related disorders, moving beyond one-size-fits-all treatment toward interventions matched to each patient’s dominant biological stress state at a given point in the disease course. However, until such validation exists, no diagnostic or therapeutic decision should be based on this framework.
